# Fracture Resistance of Endodontically Treated Teeth Restored with Preheated Short Fiber-Reinforced Composite and Preheated Composite Resin

**DOI:** 10.3390/ma18225145

**Published:** 2025-11-12

**Authors:** Semanur Özüdoğru, Sevda Tok, Mustafa Düzyol, Rahime Zeynep Erdem, Hakan Arslan

**Affiliations:** 1 Department of Pedodontics, Faculty of Dentistry, University of Istanbul Medeniyet, İstanbul 34956, Turkey; 2 Private Practice, İstanbul 34956, Turkey; 3 Department of Restorative Dentistry, Faculty of Dentistry, Istanbul Medeniyet University, Istanbul 34956, Turkey; mustafa.duzyol@medeniyet.edu.tr; 4 Department of Restorative Dentistry, Afyonkarahisar Health Sciences University, Afyonkarahisar 03030, Turkey; zeynepguvendi@hotmail.com; 5 Department of Endodontics, University of Istanbul Medeniyet, Istanbul 34956, Turkey

**Keywords:** preheated composite resin, restorative dentistry, short fiber-reinforced composite, fracture resistance, MOD cavity

## Abstract

This study aimed to assess the fracture resistance and fracture patterns of endodontically treated permanent mandibular molars restored with either preheated or nonpreheated conventional composite resin or short fiber-reinforced composite resin (SFRC). One hundred and twenty mandibular molars with prepared mesio-occluso-distal (MOD) cavities were allocated to six groups: positive control (intact teeth, no restoration, *n* = 20), negative control (endodontically treated but unrestored, *n* = 20), and four experimental groups restored with conventional composite, preheated composite, SFRC, or preheated SFRC (*n* = 20 each). After thermocycling, fracture resistance was tested using a universal testing machine at 0.5 mm/min. Data were analyzed using Jamovi software (Version 2.4.8; The Jamovi Project, Sydney, Australia). Normality was assessed with the Kolmogorov–Smirnov test. Group differences were evaluated using the Kruskal–Wallis test followed by the Dwass–Steel–Critchlow–Fligner post hoc test. The association between fracture modes and fracture strength categories was examined using the chi-square test of independence. A *p*-value < 0.05 was considered statistically significant. The positive control showed significantly greater fracture strength than all restored groups (*p* < 0.05). All restored groups had significantly higher fracture resistance than the negative control (*p* < 0.05), with no significant differences among the restored groups (*p* > 0.05). A significant association was found between fracture mode and fracture strength (χ^2^(1) = 6.97, *p* = 0.008). The preheated SFRC group showed a higher rate of restorable fractures compared to others, suggesting improved clinical reparability with preheating.

## 1. Introduction

Understanding the risk factors related to dental tissue is crucial for the success of composite resin restorations [1]. Bulk fracture, secondary caries, and microleakage are the most frequent reasons for large-restoration failure, particularly in molars that are compromised by endodontic therapy [2,3]. Enhancing restorative materials’ longevity and functionality is essential due to these challenges, which can significantly affect the ability to restore strength in endodontically treated molars with mesio-occluso-distal (MOD) cavities [4].

Recent studies have confirmed that the fracture resistance of endodontically treated teeth strongly depends on residual dentin thickness, cavity design, and restorative approach [5,6,7]. Teeth with conservative access and adequate dentin bulk demonstrate higher resistance, whereas extensive MOD cavities increase fracture susceptibility [7].

Advances in restorative materials have led to improved outcomes in such cases. Short fiber-reinforced composite (SFRC) resins incorporate short glass fibers into a polymer matrix to simulate dentin structure, enhancing load distribution and crack-deflection capacity [8,9]. Compared with conventional composites, SFRC exhibits improved mechanical integrity and energy absorption due to its fiber architecture [10,11,12].

Preheating resin-based composites before application is another promising strategy to enhance clinical performance. Preheating increases the material’s flowability and degree of monomer conversion by reducing viscosity, thereby improving polymer cross-linking and marginal adaptation [13,14,15,16]. This technique has been reported to enhance microhardness, flexural strength, and reduce polymerization shrinkage stress, potentially leading to improved restoration longevity [17,18,19,20].

Despite these developments, the combined influence of preheating and fiber reinforcement has not been comprehensively investigated [21,22]. While numerous studies have assessed preheated conventional composites or non-preheated SFRCs individually [14,15], no previous research has focused on preheated SFRC applied in endodontically treated molars with MOD cavities. The present study therefore aims to fill this gap by evaluating and comparing the fracture resistance and fracture patterns of teeth restored with preheated and non-preheated SFRC and conventional composites.

The null hypothesis was that preheating and fiber reinforcement would not significantly affect the fracture resistance or fracture mode of endodontically treated MOD molars.

## 2. Materials and Methods

### 2.1. Declaration of Ethics

This study was approved by Marmara University Ethics Committee (Approval No: 2024/55, approval date: March 2024), conducted in accordance with the Declaration of Helsinki.

### 2.2. Research Design

A power analysis realized with G*Power (Version 3.1.9.7; Germany) indicated that a sample size of 72 is necessary to achieve enough statistical power (α = 0.05, power = 0.80, effect size: 0.455) for detecting a significant effect, as per the methods described by Plotino et al. [23]. The sample size was increased to 120 teeth to ensure adequate statistical power and account for potential specimen loss during preparation or testing.

This study’s inclusion criteria required teeth that had been extracted for periodontal reasons and showed no signs of caries, wear facets, or structural defects. A stereomicroscope was used to meticulously inspect every tooth for cracks or fractures (Leica Microsystems, Wetzlar, Germany) under 20× magnification, and this study included no teeth with visible cracks or fractures.

To ensure size consistency across all the samples, measurements of each tooth’s weight, buccolingual (BL), and mesiodistal (MD) dimensions were taken. A digital micrometer and a precision scale were used for the measurements, and the permissible ranges of the 120 mandibular molars were weight (1.0–2.8 g), buccolingual width (7.0–10.8 mm), and mesiodistal width (7.5–11.2 mm). We also looked for similarities in the sizes and shapes of tooth roots. Each experimental group was assigned an equal number of teeth. Randomization of samples was performed using a computer-generated list. Teeth were then stratified and randomly assigned to groups using these parameters to ensure comparable size distribution across groups (*p* > 0.05).

All teeth were stored in distilled water at 37 °C until testing to preserve tissue hydration.

### 2.3. Specimen Preparation

Standardized MOD cavity preparations were conducted on all teeth with a consistent wall thickness of 2.5 mm and a depth of 5 mm, as guided by Forster et al. [24]. To guarantee uniformity, the same trained operator (M.D.) prepared each cavity. A digital caliper was frequently used to gauge a cavity wall’s thickness. One millimeter coronal to the cementoenamel junction (CEJ) was the gingival wall preparedness level, and the cavity walls were positioned parallel to a tooth axis. A cavity’s depth was determined using a periodontal probe (Hu-Friedy Mfg. Co., Chicago, IL, USA). Following the completion of cavity preparation, cavosurface margins were prepared in a manner perpendicular to the tooth’s surface.

To ensure consistency, root canal treatments were performed on all molars by an endodontic specialist (S.T.). A #10 stainless steel K-file (Dentsply Maillefer, Ballaigues, Switzerland) was inserted into each root canal following the removal of the pulp chamber ceiling and was maintained in position until the file tip became visible at the apex. The working length was derived by subtracting 1 mm from this length.

The root canals were prepared using Wave One Gold files (Dentsply Maillefer, Ballaigues, Switzerland). These files were used with a mild pecking motion with in and out movement with a 2–3 mm amplitude, selected based on the root canal’s structure. The device was removed after three in-and-out motions, and the procedure continued until the working length was attained; also, after irrigating a root canal with 5 mL of 2.5% sodium hypochlorite (NaOCl), a #10 K-file was used to verify apical patency. Following instrumentation, 5 mL of 17% EDTA was used to irrigate the canal for 3 min. This was followed by a final rinse with 5 mL of 2.5% NaOCl. Subsequently, sterile paper tips were used to dry the root canal.

A canal paste (AH Plus; Dentsply De-Trey, Konstanz, Germany) was prepared following the manufacturer’s guidelines, and the respective single cones were coated with the paste before to insertion into the root canal at the specified working length. Excess gutta-percha was removed with a heated plugger post-filling to avert further vertical compaction.

### 2.4. Restorative Procedure

Table 1 enumerates the restorative materials employed in this study. The teeth in the experimental groups were subjected to selective enamel etching for 30 s with 35% phosphoric acid gel (Scotchbond Etchant Gel, 3 M Espe, Saint Paul, MN, USA). This was performed subsequent to the application of a Tofflemire matrix-band system (1101C 0.035, KerrHawe, Bioggio, Switzerland) encircling the enamel surfaces. Subsequent to the etching procedure, cavities were addressed using Clearfil SE Bond (Kuraray, Tokyo, Japan) in accordance with the manufacturer’s guidelines.

The following were the tooth distributions in the experimental and control groups:

The positive control group consisted of intact teeth without cavity preparation (*n* = 20).

MOD endodontic access was prepared, but no coronal restoration was performed in the negative control group (*n* = 20).

Composite group (*n* = 20): This was performed using microhybrid composite resin (Estelite Posterior, Tokuyama Dental, Tokyo, Japan) with an incrementally applied method. The first construction of the absent proximal walls was 1 mm thick. Two millimeter-thick increments were used to repair the remaining cavity.

Preheated composite group (*n* = 20): A preheated microhybrid composite resin was applied using the same technique as the composite group.

SFRC group (*n* = 20): Absent proximal walls were constructed using a 1-mm-thick microhybrid composite resin. The SFRC (EverX Posterior, GC, Tokyo, Japan), a bulk-fill composite material designed to replicate the dentin–enamel complex, was placed in 4-mm increments and covered with a 2-mm microhybrid composite resin overlay.

Preheated SFRC group (*n* = 20): The SFRC material was preheated before application, and the cavities were restored according to the instructions provided in the SFRC group.

Each increment received 20 s of light curing at a 1 mm distance, and incremental layers were 2 mm thick.

Specimen preparation and photopolymerization were carried out at room temperature (23 ± 2 °C) for every group (except the preheated groups). The preheating temperature (54 °C) was monitored using a calibrated digital thermometer (VOCO GmbH, Cuxhaven, Germany) and maintained during transfer by placing the capsule into the cavity within 30 s of removal from the warmer. Temperature was verified using a digital thermometer connected to the Caps Warmer system to confirm constant 54 °C before each placement.

A light-curing device (Valo Cordless, Ultradent Products Inc., South Jordan, UT, USA) was employed for 20 s to achieve polymerization. The light-curing device had an irradiance of 1100 mW/cm^2^. Following restoration, the specimens were maintained in distilled water for a complete day at 37 °C. In accordance with the International Organization for Standardization (ISO) 11405 [25], thermocycling was performed for 5000 cycles, with a dwell time of 20 s and a transfer interval of 10 s, alternating between temperatures of 5 °C and 55 °C.

A thin (approximately 0.3 mm) layer of silicone impression material (Vinylight, BMS Dental, Capannoli, Italy) was applied to root surfaces to simulate the periodontal ligament. Then, to replicate alveolar bone support, each tooth was placed in self-hardening acrylic cylinders 2 mm apical to a CEJ, with the tooth’s long axis perpendicular to the base of the acrylic block.

### 2.5. Testing

A universal testing machine (Model 3345, Instron Corporation, Norwood, MA, USA) was used to conduct a fracture strength test. Force was applied perpendicular to a tooth’s long axis using a steel, 6-mm-diameter spherical tip, moving at a crosshead speed of 1 mm/min until the tooth fractured. Newtons (N) were used to record the force at the fracture. The load was applied at the central fossa of each specimen to ensure standardized contact points, and all tests were conducted in a moist environment at 37 °C.

Scotti et al. [10] proposed classifications that were used to group failure modes. Two blinded examiners (S.Ö. and H.A.) independently assessed the fracture modes under 20× magnification using a stereomicroscope (Nikon SMZ645, Tokyo, Japan), showing good inter-examiner agreement (κ = 0.91), which confirmed the reliability of the classification. If a fracture happened above a CEJ or within 1 mm of a CEJ’s apical surface, it was considered “restorable.” “Non-restorable” fractures were defined as those that were more than 1 mm apical to a CEJ [7,10].

### 2.6. Statistical Analysis

The evaluation of data was conducted using Jamovi software (Version 2.4.8; The Jamovi Project, Sydney, Australia), employing both analytical and descriptive statistical methods. A post hoc power analysis was additionally conducted using G*Power (Version 3.1.9.7), confirming that the achieved sample size maintained a statistical power greater than 0.80 at α = 0.05. One-way ANOVA was used (F (5114) = 47.90), and eta squared (η^2^ = 0.68, large) was computed as a measure of effect size. For key pairwise contrasts, Cohen’s d was additionally calculated. The normality of data distribution was evaluated using the Kolmogorov–Smirnov test, which revealed that the data were not normally distributed (*p* < 0.05). Consequently, non-parametric tests were employed for further analyses. Differences among groups were examined using the Kruskal–Wallis test and post hoc pairwise comparisons were carried out using the Dwass–Steel–Critchlow–Fligner test to identify specific group differences. In addition, a chi-square test of independence was performed to assess the relationship between fracture modes and fracture strength categories. Fracture strength values were divided into two groups (low vs. high) based on the median value (1638.30 N). For all statistical analyses, a significance level of *p* < 0.05 was adopted.

## 3. Results

**Figure 1:**
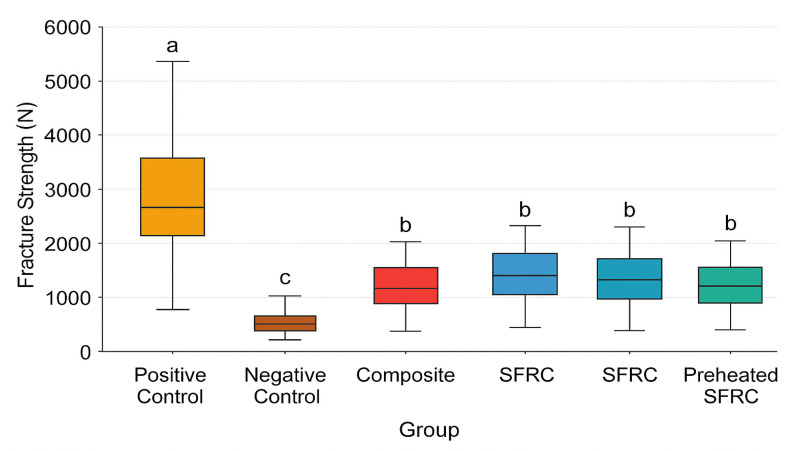
Mean fracture strength (N) of all experimental groups. *Y*-axis represents fracture strength in Newtons (N), and *X*-axis shows the six groups (Positive Control, Negative Control, Composite, SFRC, Preheated Composite, and Preheated SFRC). Different superscript letters (a, b, c) show statistically significant differences among groups (*p* < 0.05) SFRC: short fiber-reinforced composite.

The average fracture resistance values (Newtons) and standard deviations for each group are presented in Table 2 and Figure 1. The ANOVA revealed significant differences among the groups (*p* < 0.05, η^2^ = 0.68, large). The positive control group exhibited significantly superior fracture strength compared to the composite, preheated composite, SFRC, and preheated SFRC groups (*p* < 0.05). The fracture strength of the negative control group was significantly lower than that of the other groups (*p* < 0.05). The fracture strengths of the SFRC, preheated SFRC, composite, and preheated composite groups exhibited no significant differences.

**Figure 2:**
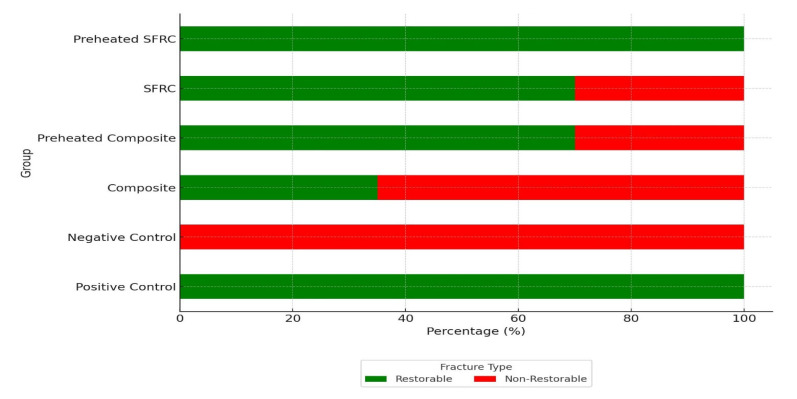
Distribution of fracture types among groups. The bars indicate the percentage of restorable and non-restorable fractures for each group. Restorable fractures were defined as those above or within 1 mm of the cementoenamel junction (CEJ), while non-restorable fractures were those extending more than 1 mm apical to the CEJ.

**Figure 3:**
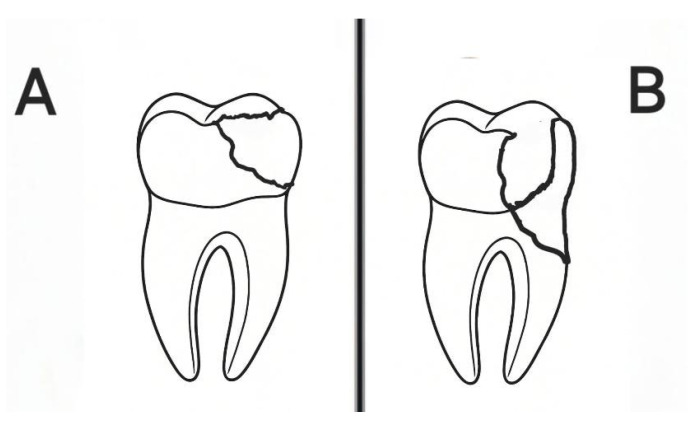
Schematic representation of fracture types: (**A**) Restorable fracture, (**B**) Non-restorable fracture.

For each group, Figure 2 shows the frequency of fractures that could be repaired and those that cannot. The SFRC and preheated composite groups (70%) and the composite group (35%), in order of decreasing proportion, had the highest percentage of restorable fractures in the preheated SFRC group (100%). All fractures were classified (Figure 3) as non-restorable in the negative control group, and the lowest percentage of restorable fractures was noted. A significant association was found between fracture mode and fracture strength (χ^2^(1) = 6.97, *p* = 0.008). Non-restorable fractures were more frequently observed in specimens with higher fracture strength values, while restorable fractures predominated in the lower strength category.

## 4. Discussion

Endodontic treatment is commonly known to increase the risk of tooth fracture, especially in teeth with large MOD cavities [24,25,26]. Limited data regarding using preheated SFRC or preheated composite resin to enhance teeth repair with significant coronal damage are available in the literature. Therefore, this present study aimed to assess the efficacy of preheated SFRC by comparing the fracture strength of endodontically treated teeth with MOD cavities.

In this study, teeth that were still intact showed better fracture resistance than the composite, SFRC, preheated composite, and preheated SFRC groups. The null hypothesis was rejected because, despite no discernible differences between the experimental groups, all exhibited greater fracture resistance than the negative control group.

Several coronal treatment options are available for teeth that have undergone endodontic therapy [27,28]. The components of SFRC are fillers made of inorganic particles and short glass fibers arranged randomly inside a semi-interlocking polymer network matrix. A short fiber structure improves a restoration’s durability by giving it dentin-like hardness and transmitting strain to the fibers via the polymer matrix [29,30].

Research conducted by Yasa et al. [5] and Atalay et al. [4] assessed the impact of fiber-reinforced composites on the fracture resistance of endodontically treated teeth, revealing that these composites provide fracture resistance comparable to that of traditional composite resin alone. These results align with the findings of the current investigation. Likewise, Fráter et al. [15] observed no statistically significant enhancement in the fracture resistance of molar teeth with MOD cavities when SFRC was employed. Eapen et al. [26] discovered that premolars that had undergone endodontic treatment and were repaired with SFRC showed better fracture resistance than premolars restored only with composite resin.

The differences between the aforementioned studies may be that they were conducted on different tooth groups, and the samples were not distributed equally to groups in terms of standardized weight or MD and BL dimensions (as in this current study); instead, they were distributed randomly and only standardized MD and BL dimensions were applied. Ertaş et al. [27] highlighted that specimen weight or volume is statistically more significant than MD and BL dimensions when establishing groups to assess fracture resistance.

Preheating composite resins has been demonstrated to enhance fluidity, decrease microleakage, and favorably affect mechanical qualities. Furthermore, it has been discovered that preheating resins to 54 °C or 60 °C increases the degree of conversion, hardness, and fracture toughness [19,31,32,33]. The outcomes of this current study are consistent with those of Gade et al. [21] and Othman and Zainab et al. [22], who found no discernible variation in fracture resistance between preheated composite resins and conventional composite resins or intact teeth.

Almeida et al. [31] demonstrated that preheating SFRC to 60 °C enhances its flexural strength and microhardness by reducing viscosity, improving fiber–matrix interaction, and decreasing void formation. This improves stress transmission and interactions between fibers and a resin matrix. Preserving residual tooth structure is crucial for teeth that have been endodontically treated, particularly in a cervical area. Additionally, Lempel et al. [16] showed that increasing the degree of conversion of a fiber-reinforced composite by preheating it to 55 °C was beneficial. Similarly, in our study, preheating to 54 °C was done, and the preheated groups showed a higher rate of fracture that could be restorable than the nonpreheated groups. Preheating the composites before photopolymerization may have improved fracture behavior by lowering viscosity and improving flowability by raising the degree of conversion [32]. A denser cross-linked polymer structure was produced when the temperature rose due to an increase in radical and monomer mobility [21,33]. In addition, numerous other studies have shown that preheating improves bond strength to dentin and polymer cross-linking efficiency, which may contribute to better stress distribution under load [19,33].

Finite-element analyses have also been employed to evaluate the fracture behavior of restored teeth under standardized stress conditions [11,34]. These computational findings support the in vitro results by confirming that fiber reinforcement and improved interfacial bonding can effectively reduce cusp deflection and internal stress concentration in MOD cavities.

The present study expands on previous literature by exploring the combined influence of preheating and fiber reinforcement—an aspect that has received little attention in the existing body of evidence. Clinically, this suggests that using preheated SFRC may promote more restorable fracture patterns, potentially improving the prognosis of endodontically treated posterior teeth.

However, several limitations should be acknowledged. First, this is an in vitro study that cannot fully replicate intraoral conditions, such as thermal fluctuations, occlusal fatigue, and humidity. Second, only two restorative materials and one mechanical property (fracture resistance) were examined; parameters such as dentin bond strength, microleakage, cyclic fatigue, and adhesive interface integrity were not assessed. Third, all procedures were performed by a single operator, which, despite ensuring consistency, could introduce operator bias. Lastly, finite-element or fatigue testing could further substantiate these findings in future investigations.

Despite these limitations, the results provide valuable preliminary insight into how preheating and fiber reinforcement may influence both fracture strength and fracture pattern, paving the way for subsequent experimental and clinical validation.

## 5. Conclusions

The fracture resistance of the preheated and nonpreheated groups did not differ significantly. The preheated SFRC showed better fracture behavior than the nonpreheated groups. Preheated SFRC may be preferred when restorable fracture patterns are desired. However, further research, including similar experimental settings, is needed to reveal the importance of preheating.

## Figures and Tables

**Table 1:** Details of materials used in this study.

Material	Type	Composition	Manufacturer	Batch No
Scotchbond Etchant Gel	Etching agent	Phosphoric acid, synthetic amorphous silica, water	3M ESPE, St. Paul, MN, USA	N414370
Clearfil SE Bond	Bonding agent	Primer: MDP, HEMA, hydrophilic dimethacrylate, photo-initiator, water Bond: MDP, HEMA, Bis-GMA, hydrophobic dimethacrylate, photo-initiators, silanated colloidal silica	Kuraray, Tokyo, Japan	C60001
Estelite Posterior	Micro-hybrid composite	Bis-GMA, TEGDMA, Bis-MPEPP	Tokuyama Dental, Tokyo, Japan	243E67
Ever X Posterior	Short fiber-reinforced composite	Bis-GMA, PMMA, TEGDMA, Salinated E-glass Fiber, Barium Glass	GC Corporation, Tokyo, Japan	2003061

**Table 2:** Mean fracture resistance values (Newtons, N) of all the sample groups.

Groups	N	Mean ± SD (N)	Median (25th–75th Percentile)
Positive Control	20	2598.72 ± 863.76	2377.0 (1897.3–3418.1) ^a^
Negative Control	20	299.59 ± 98.50	270.3 (229.7–382.1) ^d^
Composite	20	1596.95 ± 303.54	1606.0 (1416.3–1812.2) ^b^
SFRC	20	2020.85 ± 551.30	1897.7 (1581.3–2393.5) ^b^
Preheated Composite	20	1648.21 ± 322.11	1555.6 (1378.1–1922.5) ^b^
Preheated SFRC	20	1668.84 ± 423.34	1528.1 (1303.5–1973.1) ^b^

Values are expressed as median (25th–75th percentile). Different superscript letters indicate statistically significant differences among groups (*p* < 0.05, Kruskal–Wallis test, post hoc Dwass–Steel–Critchlow–Fligner) SD: Standard Deviation; SFRC: short-fiber reinforced composite.

## Data Availability

The original contributions presented in this study are included in the article. Further inquiries can be directed to the corresponding author.

## Abbreviations

SFRC	Short Fiber-Reinforced Composite
MOD	Mesio-Occluso-Distal
CEJ	Cementoenamel Junction
ANOVA	Analysis of Variance
